# Inflammation with HTLV-1-specific CTLs occurs in the spinal cord of HTLV-1 carriers and the brain of the patients with HAM/TSP

**DOI:** 10.1186/1742-4690-11-S1-P7

**Published:** 2014-01-07

**Authors:** Eiji Matsuura, Satoshi Nozuma, Toshio Matsuzaki, Osamu Watanabe, Ryuji Kubota, Shuji Izumo, Hiroshi Takashima

**Affiliations:** 1 Department of Neurology and Geriatrics, Kagoshima University Graduate School of Medical and Dental Sciences, Kagoshima, Japan; 2 Department of Molecular Pathology, Center for Chronic Viral Diseases, Kagoshima University, Kagoshima, Japan

## 

Recently, a study by Morgan et al. identified that the white matter lesions are detectable with MRI study in HTLV-1 carriers at the same frequency as HAM/TSP patients. Little is known about the nature of the brain lesion and its relation to the spinal cord lesion. We assessed the inflammatory change including HTLV-1-specific cytotoxic T lymphocytes in the brain lesion of the patient with HAM/TSP, in the spinal cord of HTLV-1 carrier immunohistochemically. Fresh frozen samples including one brain from the patient with HAM/TSP and four spinal cords from HTLV-1 carriers were for use in this study. Inflammatory cells in the sections of the brain and spinal cords were stained with anti-CD4, anti-CD8 antibodies. HTLV-1-specific CTLs were detected with either phycoerythrin (PE)-conjugated HLA-A*0201/Tax11-19 tetramer or HLA-A*2402/Tax301-309 tetramer, and followed by rabbit anti-PE antibody as the secondary antibody. The signals from the third antibody with fluorescence were captured by confocal laser scanning microscopy (CLSM). HTLV-1-specific CTLs were frequently detected in the brain lesion of the patient with HAM/TSP. The CTLs were also detected in all the spinal cords of four HTLV-1 carriers. Interestingly, inflamed lesions of HTLV-1 carrier were distributed mainly in the lower thoracic level of the spinal cords like the spinal cord of HAM/TSP. These results suggest that inflammatory changes occur simultaneously in the spinal cord and the brain of the patients with HAM/TSP, and that latent inflammatory change may occur in asymptomatic HTLV-1 carriers.

